# Complex pelvic fracture with massive hemorrhage in low resource settings: Case report

**DOI:** 10.1016/j.radcr.2023.08.043

**Published:** 2023-09-07

**Authors:** Jeannot Baanitse Munihire, Hauwa Shitu Balarabe, Anthony Ayotunde Olasinde, Joshua Muhumuza

**Affiliations:** a Faculty of Clinical Medicine and Dentistry, Department of Surgery, Kampala International University Western Campus, Ishaka-Bushenyi, Uganda; b Faculty of Medicine, General Surgery department, Université Catholique la Sapientia de Goma, and Charite Maternelle Hospital, Goma, Democratic Republic of the Congo; c Department of Orthopaedic Surgery and Traumatology, Federal Medical Centre, Owo Ondo State, Nigeria

**Keywords:** Pelvic fracture, Bladder rupture, Vascular injury, Mortality associated

## Abstract

Pelvic fractures can range from simple, requiring almost no therapy, to complex, mandating the attention of the orthopedic surgeon, trauma surgeon, interventional radiologist, or other specialists because they are associated with multisystem injury and life-threatening hypotension. We present a 16-year-old male who presented with a complex pelvic fracture following a motor vehicle accidents that did not survive despite the efforts in resuscitation. In complex pelvic fracture with hemodynamic instability (hypotension persevered) and high index suspicion of bladder injury, there is an immediate need for operative intervention, regardless of negative/positive FAST (Focused Assessment with Sonography in Trauma).

## Introduction

Pelvic ring disruption is a significant cause of morbidity and mortality in polytrauma patients with a high rate of associated injuries. Because of the vital force necessary to disrupt the pelvic ring in young patients, it is not surprising that up to 80% of these patients also have additional musculoskeletal injuries. Mortality rates vary from 15% to 25%, resulting from uncontrolled hemorrhage or other associated injuries [1].

Pelvic fractures are the third most common cause of death associated with motor vehicle accidents after central neurological system and chest injuries [2]. Mortality increases almost 13-fold when the patient is hypotensive. In combination with a head or abdominal injury requiring surgical intervention or an open pelvic fracture, mortality increases to 50%. Mortality approaches 90% when both procedures are necessary (intervention on the head and the pelvis) [3].

Therefore, an immediate and aggressive approach is required for diagnosis and treatment in emergency services to minimize mortality and morbidity [2]. Excluding oliguria, anuria, and hematuria after pelvic trauma is always essential, as well as hemorrhage in the urethral meatus or urethral catheter, since these indicate urinary tract injury. Timely intervention in genitourinary injuries prevents complications such as impaired renal function, urinary incontinence, sexual dysfunction and deaths [4]. Patients who present with hemodynamic instability continue to be extremely challenging to treat [5].

We present a patient with a complex pelvic fracture complicated with a massive hemorrhage in a low-resource setting hospital.

NB:

• Low resource setting hospital is a facility where basic, essential equipment and materials for advanced resuscitation are lacking. For instance, the blood transfusion requirement of this patient could not be met because type-specific blood got exhausted. We later used uncross-matched, non–type-specific blood.
• Our facility is less than a level 1 trauma center in the United States.
• A CT scan was not done for the patient because he was hemodynamically unstable. The CT scanner is a separate unit in the hospital.

## Presentation of case

A 16-year-old male arrived at the accident and emergency unit following a head-on collision with a speeding car while riding a bicycle. The patient was alert and slightly responsive at the scene. He received over 2000 mL of crystalloid in a nearby medical facility 3h30 minutes prior to the presentation at the KIU-TH emergency unit, where resuscitation was continued.

Upon arrival, the patient had a blood pressure of 92/78 mmHg, a respiratory rate of 28 breaths/min, a peripheral O2 saturation of 98%, and a heart rate of 130 beats per minute. Glasgow coma score of 15/15, the patient was awake and slightly alert, with conjunctival pallor. Bilateral cubital, large bore intravenous cannula gauge 16 was inserted in a large forearm vein, and he had 1 units of type O-negative blood transfused. Intramuscular tetanus toxoid 0.5 mL was given. The patient's airway was intact, with tachypnea (respiratory rate 28), tachycardia and slight lower abdominal swelling but no tenderness.

On musculoskeletal exam, bruises were on the left lateral thigh, lower back, and right lumbar region. The patient had tenderness on slight pelvic compression and palpation of L2-L3. There was a palpable swelling in the sacral region. One urethral catheterization attempt resulted in free blood drainage into the urinary bag, and the catheter was clamped and removed immediately. The left leg was cold, mottled and pulseless, with complete paralysis and loss of sensation.

**Fig. 1:**
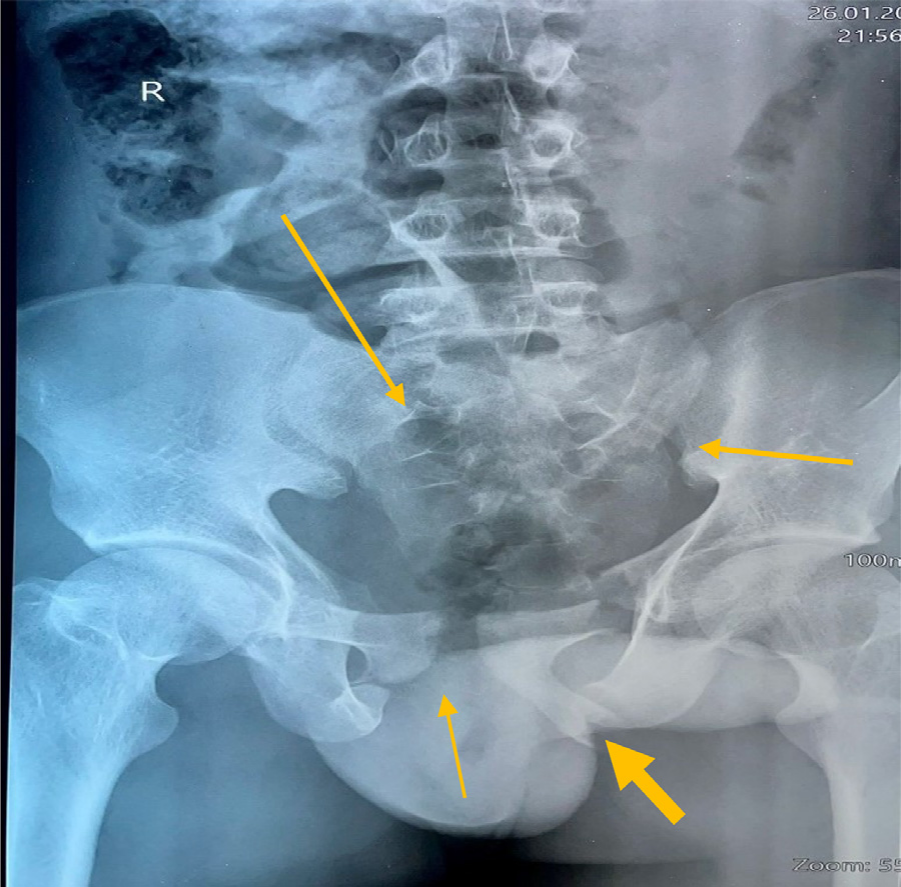
Complex pelvic fracture with widening and displacement of the pubis rami and widening of the S1joints bilaterally: arrows shows multiple fractures in the pelvis.

Emergent imaging exams: The chest x-ray was normal, abdominal USS revealed intraperitoneal hemorrhage, and the pelvic x-ray revealed a complex pelvic fracture with widening symphysis pubis (APC 11 and bilateral vertical shear). Fig. 1 shows complex pelvic fracture with wide public symphysis and displacement of the pubis rami.

**Fig. 2:**
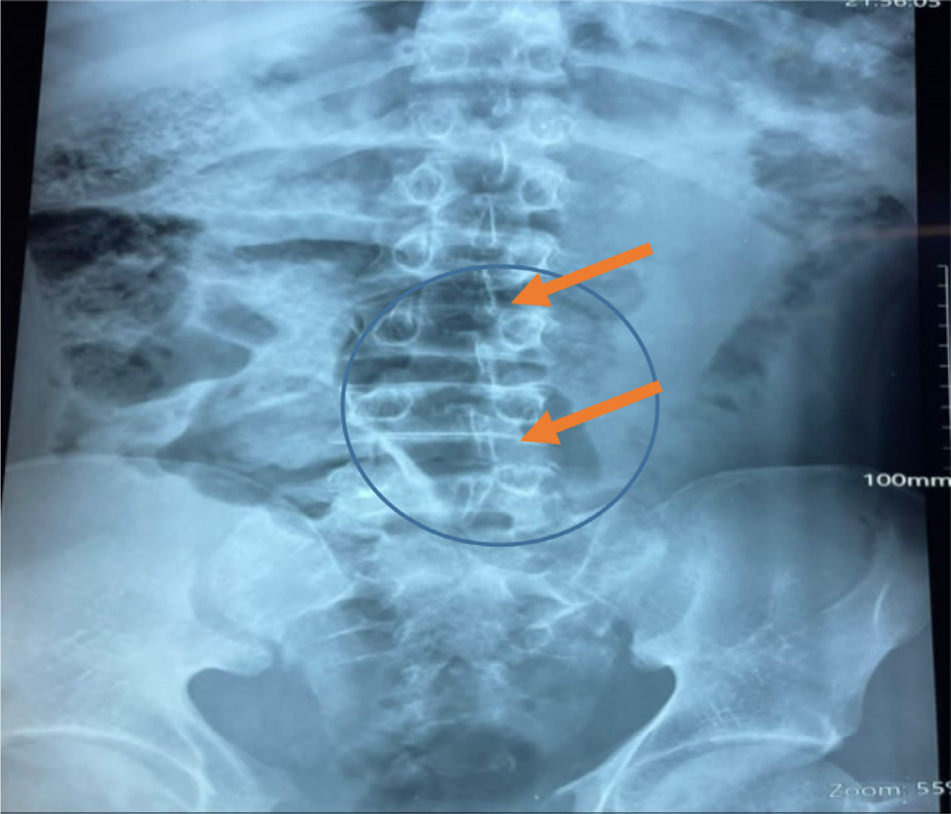
Fracture on the lumber spinal column at L2-L3 and L5.

On the vertebral column, there was a fracture at L2-L3 with reduction in vertical height of L3 (Fig. 2 shows fracture on the vertebral column at L2-3). A lateral view could not be done because of the patient's critical status.

Improvised pelvic binder made from bed sheets was wrapped around the patient's pelvis. Another two 2 units of O-negative packed red blood cells were administered. The patient's systolic pressure dropped to 68 mmHg and he developed progressive abdominal distention in the face of continued hypotension. Patient was planned for emergent laparotomy; however he became unresponsive, and resuscitation was unsuccessful, and died in the emergency room. From the first facility to arrival at the KIU-TH emergency unit he spent 3 hours and in our facility he spent 2 hours before dying due to unresponsiveness to continued resuscitation. Unfortunately, the autopsy was not done because the relative declined consent.

## Discussion

Pelvic fractures are direct or indirect outcomes of anteroposterior compression, lateral compression, vertical shear, or a combination of these 3 depending on the direction of a high-energy force. These injuries lead to high morbidity and are often accompanied by injuries to the bladder and urethra [2]. According to Burgess and Young's 1990 classification system of pelvic fracture, which is based upon the mechanism of injury and associated injuries and their severity [5], our patient presented a complete disruption anteriorly and posteriorly, displacement of vertical pelvic rami, vertical fractures of all rami and vertical instability. This patient appears to have a combined mechanism of injury, an APC-III (anteroposterior compression type 3) and bilateral Vertical shear type fracture.

These pelvic fractures are high-impact injuries with mortality rates between 5% and 33% [5,6]. The commonly occurring associated injuries include: intracranial (40%-66.1%), splenic (9.3%-37%), colorectal (6.8%-29.1%), bladder (2.5%-28%), chest (6%-16.6%), liver (5.6%-19%), lower limb fracture(s) (17%), pulmonary (9.3%), upper limb fracture(s) (3%) and diaphragmatic rupture (3%-21%) [6,7]. In our case, the bladder and the vertebral column were associated.

Pelvic fractures with concomitant lower urinary tract injuries are associated with high morbidity and mortality, and early diagnosis and appropriate management in such cases play a significant role in the patient's prognosis [7].

The indicative signs of bladder rupture include a more than 1-cm diastasis of the symphysis pubis and a displaced fracture of more than 1 cm involving the obturator ring [8]. Frank hematuria is also associated with bladder rupture in 16%-27% of patients who sustain a pelvic fracture, regardless of radiological findings [7]. However, classical findings may be absent in 29%-76%, and a high index of suspicion should be maintained [6]. A 1-time gentle attempt at urethral catheterization is reasonable, even if blood is seen at the meatus. If the catheter fails to pass easily or drain clear urine, it should be removed immediately [4,6].

As in the case of our patient, there was no visible blood on the urethral meatus, so 1 attempt catheterization was done; however, there was free active drainage of frank hemorrhage, and the catheter was clamped and removed afterwards, together with the radiological findings which confirm our suspicion of bladder rupture with concomitant vascular injury.

Most bladder ruptures in pelvic fractures can lead to either an extraperitoneal or intraperitoneal extravasation; however, severe injuries can co-occur. Some cases of extraperitoneal injuries can be managed conservatively [7]. Contrary to this, intraperitoneal bladder ruptures almost always require immediate surgical intervention to minimize the occurrences of intraperitoneal contamination [7,9]. The simultaneous occurrence of intraperitoneal and extraperitoneal bladder injuries is uncommon and is associated with a higher death rate [10].

The biggest question is what steps could have been taken in a low-resource setting to improve this patient's outcome. The initial plan was to take the patient for an explorative laparotomy for pelvic packing. If such a patient is to be taken to the operating theatre, then bilateral internal iliac artery ligation should be considered in the presence of massive venous bleeding [5,11]. Existing literature has less sampling of patients with pelvic fractures who have undergone this procedure [5].

With a negative FAST (Focused Assessment With Sonography In Trauma) in the accident and emergency room, one could argue that the CT scans of the abdomen and pelvis were not needed. Instead of the CT scanner, the patient could be taken to interventional radiology to undergo an angiogram with possible bilateral temporary internal iliac embolization [12].

In low resources settings, some imaging (X-ray, sonography) are always done in the day and not available in many hospitals, others are rare and expensive like CT Scan, interventional radiology, angiography or MRI. With this case report, FAST negative or positive, we suggest to do explorative laparotomy for possible pelvic packing or internal iliac ligation in case of urinary bladder rupture, external fixation or stabilization for multiple fracture or displaced fractures, and mainly if hypotension persevere (patient remains unstable hemodynamically). Despite the hemodynamic stability, explorative laparotomy is indicated if pelvic fracture with and signs of bladder rupture.

## Take note

Images were taken with camera phone and that is only resolution that we have.

## Ethical approval

Ethical approval is exempt/waived at our institution.

## Research registration

Not applicable.

## Provenance and peer review

Not commissioned, externally peer-reviewed.

## Authors’ contributions

JBM and SHB managed the patient and wrote the first draft. AAO and JM edited and reviewed the paper. All authors read and approved the final version to be published.

## Patient consent

Written informed consent was obtained from the patient's parents/legal guardian for publication of this case report and accompanying images. A copy of the written consent is available for review by the Editor-in-Chief of this journal on request.

## References

[1] Smith W, Williams A, Agudelo J, Shannon M, Morgan S, Stahel P (2007). Early predictors of mortality in hemodynamically unstable pelvis fractures. J Orthop Trauma.

[2] Dinçer R, Öztürk S.A (2021). Urological complications in pelvic fractures: correlation between types of fractures and urinary injuries. Isparta, Turk J Health Sci Life.

[3] MacKenzie EJ, Bosse MJ, Kellam JF, Pollak AN, Webb LX, Swiontkowski MF (2006). Early predictors of long-term work disability after major limb trauma. J Trauma.

[4] Anderson RE, Keihani S, Moses RA, Nocera AP, Selph JP, Becerra CMC (2020). Current management of extraperitoneal bladder injuries: results from the multi-institutional genito-urinary trauma study (MiGUTS). J Urol.

[5] Knight CJ., Mboumi IW, Thompson EC (2017). Severe pelvic fracture with profound hypotension: a case report and treatment algorithm. J Surg Case Rep.

[6] Barratt RC., Jason BA, Mundy R., Tamsin GW (2018). Pelvic fracture urethral injury in males—mechanisms of injury, management options and outcomes. Transl Androl Urol.

[7] Alfayez SM, Allimmia K, Alshammri A, Serro F, Almogbel R, Abdullah BD (2016). Urological injuries associated with pelvic fractures: A case report of a detached bone segment inside the bladder. Int J Surg Case Rep..

[8] Avey G., Blackmore C.C., Wessells H., Wright J.L., Talner L.B. (2006). Radiographic and clinical predictors of bladder rupture in blunt trauma patients with pelvic Fracture. Acad Radiol.

[9] Muneer M., Abdelrahman H., El-Menyar A., Zarour A., Awad A., Al-Thani H. (2015). Spontaneous atraumatic urinary bladder rupture secondary to alcohol Intoxication: a case report and review of literature. Am J Case Rep.

[10] Myers JB, Hotaling JM, Brant WO, Enniss TM (2015). Trauma and reconstruction management of a case of severe pelvic fracture related bladder trauma. Urol Case Rep.

[11] Velmahos GC, Mattox KL, Moore EE, Feliciano DV (2013). Trauma.

[12] Velmahos GC, Toutouzas KG, Vassiliu P, Sarkisyan G, Chan LS, Hanks SH (2002). A prospective study on the safety and efficacy of angiographic embolization for pelvic and visceral injuries. J Trauma Acute Care Surg.

